# Decision Tree-Based Classification for Planetary Gearboxes’ Condition Monitoring with the Use of Vibration Data in Multidimensional Symptom Space

**DOI:** 10.3390/s20215979

**Published:** 2020-10-22

**Authors:** Piotr Lipinski, Edyta Brzychczy, Radoslaw Zimroz

**Affiliations:** 1 Computational Intelligence Research Group, Institute of Computer Science, University of Wroclaw, 50-383 Wroclaw, Poland; lipinski@cs.uni.wroc.pl; 2 Faculty of Mining and Geoengineering, AGH University of Science and Technology, 30-059 Cracow, Poland; brzych3@agh.edu.pl; 3 Faculty of Geoengineering, Mining and Geology, Wroclaw University of Science and Technology, 50-421 Wroclaw, Poland

**Keywords:** planetary gearbox, condition monitoring, vibration, spectral analysis, non-stationary operations, multidimensional symptom space, decision trees

## Abstract

Monitoring the condition of rotating machinery, especially planetary gearboxes, is a challenging problem. In most of the available approaches, diagnostic procedures are related to advanced signal pre-processing/feature extraction methods or advanced data (features) analysis by using artificial intelligence. In this paper, the second approach is explored, so an application of decision trees for the classification of spectral-based 15D vectors of diagnostic data is proposed. The novelty of this paper is that by a combination of spectral analysis and the application of decision trees to a set of spectral features, we are able to take advantage of the multidimensionality of diagnostic data and classify/recognize the gearbox condition almost faultlessly even in non-stationary operating conditions. The diagnostics of time-varying systems are a complicated issue due to time-varying probability densities estimated for features. Using multidimensional data instead of an aggregated 1D feature, it is possible to improve the efficiency of diagnostics. It can be underlined that in comparison to previous work related to the same data, where the aggregated 1D variable was used, the efficiency of the proposed approach is around 99% (ca. 19% better). We tested several algorithms: classification and regression trees with the Gini index and entropy, as well as the random tree. We compare the obtained results with the K-nearest neighbors classification algorithm and meta-classifiers, namely: random forest and AdaBoost. As a result, we created the decision tree model with 99.74% classification accuracy on the test dataset.

## 1. Introduction

The planetary gearbox (PG) is a crucial element of many mechanical systems. Requirements related to the high power to be transmitted, high transmission ratio, and small dimensions/weight cause planetary gearboxes to be complex, and they are utilized in advanced systems (helicopters, wind turbines, mining machines, etc.), so there is a need to ensure their reliability during operation. Damage detection in PGs is also crucial, and one can find many interesting techniques developed in recent years.

A general layout for condition monitoring is to pre-process the signal, extract features, and classify them, often with the use of advanced data analysis techniques. Most of the diagnostic procedures can be divided into two groups: the first one focuses on an advanced signal pre-processing/feature extraction and uses a simple decision scheme, and the second one is related to a set of simple features and advanced data (features) analysis by using artificial intelligence.

The selection of the technique depends on the damage type (localized damage, i.e., crack, spall, pitting, breakage of a single tooth, or distributed damage).

For localized damage, the most important issue is to extract a weak impulse signal from the noisy observation by advanced signal filtering or modeling. One may find works related to: signal filtering, extraction, decomposition [1,2,3,4,5,6,7], signal modeling [8,9,10], multivariate statistics [11], advanced signal processing for time-frequency or bispectral representation [12,13], and other advanced techniques [14,15,16].

For a distributed change of condition, energy-based features are usually used [17,18]. They can be defined in the time domain [18] as the RMS, peak-to-peak, etc., or the energies/amplitudes of particular spectral components or frequency bands [19]. A review can be found in [20,21,22].

In the second stage (when the signal is pre-filtered (de-noised) and one is able to obtain a representative set of diagnostic features), most of the applications exploit advanced data analysis techniques (so-called data mining). In many cases, due to the complexity of the machine, the detection/diagnosis process is performed in the multidimensional symptom space (mDSS) [23]. In the paper, this approach is explored. The mDSS can be constructed from various types of data (load, speed, energy of vibration etc.) or as we have here: a family of parameters describing the same component in the same domain (amplitudes of spectral components related to planetary stage mesh frequencies). An application of decision trees for the classification of spectral-based 15D vectors of diagnostic data is proposed. Spectral analysis is one of the most frequently used in engineering practice. An explanation of the feature extraction phase will be minimized, and we will refer to [24,25].

Usually, diagnostic data are redundant and require some pre-processing that allows us to reduce the data dimensionality and classify the data in a smaller space. Therefore, there are different techniques used for feature compression, grouping, and classification. The most popular is to use singular value decomposition (SVD) or principal component analysis (PCA) as a method for data dimension reduction [23,26,27,28,29,30]. Data transformation techniques as PCA will design a new (with less dimension) set of features. Unfortunately, this means the link to the physical interpretation of these variables is lost (Principal Component No. 1 is no longer the energy of the spectral component). Thus, the selection of informative variables is an interesting approach. In [29], a novel hybrid approach was applied, namely multivariate linear regression (MLR) and variable shrinkage by the least absolute selection and shrinkage operator (Lasso). In the case of non-linear dependencies between features, the other multidimensional approaches can be applied like kernel principal component analysis (KPCA) [31,32,33,34] or t-distributed stochastic neighbor embedding (t-SNE) [35].

When data related to both bad and good conditions are available, a so-called artificial neural network is used [36,37,38,39,40,41]; see for a review [42]. Most of the techniques try to apply known algorithms to condition monitoring and create diagnostic procedures using classifiers such as support vector machines, multi-layer perceptron neural-network-based systems, self organizing maps (Kohonen network), etc. The center of gravity is how to apply AI tools for condition monitoring rather than how to design a new, universal classification algorithm (in general). Interesting examples of soft computing might be found in [43,44], among others.

Decision trees (DTs) are well known in the data mining community; however, they are not frequently used in diagnostic applications. It will be shown that DTs may be used for classification, as well as feature selection for data dimensionality reduction. It should be highlighted that opposite to PCA/SVD, the DTs select features, not transform them. This is advantageous during the results’ interpretation. When only data in good condition are available, outliers’ analysis can be used as a powerful tool for a one-class classification [45]. A review can be found in [46].

In this paper, we will focus on a distributed form of a change in condition. We will extend the concept presented in [17], where it was suggested that by the monitoring of the amplitudes of spectral components related to planetary gearbox mesh frequency concerning operating conditions (instead of monitoring diagnostic features only), it is possible to significantly improve the probability of correct diagnosis (up to 80%) of the complex planetary gearbox used in mining machines operating under a time-varying load (TVL). Using information related to TVL, the authors discovered a relation between TVL and the values of spectral components, and they have took advantage of this dependency as a new diagnostic feature. It should be noted that in comparison to the classical statistical analysis of features, the method proposed in [17] was very successful. The authors of [17] were able to recognize the faultless condition of a gearbox for proper load conditions.

However, this approach failed in the case of the unloaded gearbox (on average, 20% of the analyzed data were acquired under small load or no load conditions). In this paper, we exploit the multidimensionality of diagnostic data, and we will propose several advanced techniques that, as will be shown later, are able to classify observations for a whole load range, moreover without information about the load.

In this paper, we propose an efficient method of classifying planetary gearboxes’ diagnostic data based on decision trees and adaptive learning algorithms like classification and regression trees (CART) with the Gini index and entropy [47], as well as the random tree algorithm (RT) [48]. We compare the obtained results with the K-nearest neighbors (KNN) classification algorithm and meta-classifiers, namely: random forest (RF) and AdaBoost (ADA).

Although decision trees have proven their capability in the efficient classification of rotating machinery and vibration data [49,50,51], the efficiency of such a classification strongly depends on data characteristics, especially their dimensionality and correlations, as well as the pre-processing method and the learning algorithm. It should be noted here that despite the many intelligent techniques described in the literature, there are just a few related to decision trees, and none of them concerns planetary gearboxes in non-stationary operation.

In the proposed approach, the condition of the planetary gearbox is described by a 15-dimensional features vector, and decision trees are built based on these 15 features, extracted earlier from the original vibration signal via spectral analysis. It should be clarified that we did not use spectral analysis for raw non-stationary signals. Before spectral analysis (SA), a particular procedure of signal segmentation was applied. As a result of this, the SA was applied to locally stationary segments of a non-stationary (in general) signal. The length of the segment comes from the kinematics of the system. It is equal to the length of the digging cycle related to bucket wheel excavator operation. However, it is not a critical value, because the most significant variability of the signal is low frequency. More information will be provided in the next section. It will be shown that by the application of the classification algorithm (decision trees) in the multidimensional symptom space (MDSS, as suggested by Cempel [23]), it is possible to improve the efficiency of classification up to 99% (this means that we are able to recognize the condition even if data are acquired for a “no load”/ “small load” case). Instead of the sum of the components’ amplitudes, we will use an array of components’ amplitudes, and we will exploit not only changes in the amplitudes, but also the relation between them for different technical states and load values, i.e., the behavior of data in the multidimensional space.

The original contributions of this paper are related to the obtained results, and they may be defined as: (i) the usage of multidimensional spectral data instead of aggregated ones may significantly improve classification efficiency, even without any information about the non-stationarity of the operating conditions; (ii) using DT for 15D data allows recognizing two classes with very high efficiency; and moreover, (iii) DT allows extracting simple decision-making rules (it then else type) and threshold values for them. It could be directly implemented in a condition monitoring system.

It will be shown that the application of the decision trees algorithm is quite simple; however, one must be aware that despite such a simple formulation, many issues must be addressed in order to obtain high classification efficiency. Firstly, the correct training datasets must be used in the learning process. This means that proper representation of two classes should be selected to train the structure. Secondly, applying different learning algorithms may result in different decision trees: some algorithms usually lead to deeper trees, others to wider ones, etc. Thirdly, correct values for the parameters of the learning algorithm must be set. Finally, in order to improve classification efficiency, proper constraints concerning the structure of the tree must be applied to avoid overfitting.

All computation described in this paper was performed with Python [52], which is a common data mining tool with open source on the GPL license, widely used in the data mining community. It enables us not only to perform the data analysis manually, but also to integrate it with any external enterprise software written in Java.

The paper is structured as follows: In Section 2, an introduction to the problem formulation and data description are provided. The obtained results and discussion are presented in Section 3. Finally, Section 4 concludes the paper and provides several directions for future work.

## 2. Methodology

### 2.1. Problem Formulation and Data Description

As input data for classification, we used the same data as in [17,28,29]. Just to recall some basic information, the MDSS consists of 15 variables. M is the cases describing a good condition, and N is the cases describing the bad condition (M = 951, N = 1232). Variables that constitute MDSS come from spectral analysis of the vibration signal. As a result of vibration measurement, one gets time series analysis with discrete time. There are many time domain diagnostic features; however, the engineering community prefers to use the frequency domain. To transform the raw signal into the frequency domain, fast Fourier transform (FFT) is used. It is basic knowledge currently, so we will skip the mathematical foundation of this. Spectral analysis (SA) is one of the most popular techniques for vibration data processing because the analysis of frequency content allows finding a physical link to the rotating parts of the machine [17], and it has found many applications [11,24,53,54]. One of the most critical limitations of SA is the requirement regarding the stationarity of the signal. As was mentioned before, we are focusing on machine diagnostics under non-stationary operating conditions; therefore, applying SA to a raw vibration signal is not possible. To avoid these constraints, we use the segmentation of the time series, assuming that during the segment, the signal is locally stationary. The segment length used here is related to the cycle of digging (excavating the material by the bucket wheel excavator). It was found that the non-stationarity of the signal for a ca. T = 1s segment duration is negligible due to the low-frequency nature of the load fluctuation.

To find parameters of the signal pre-processing, the mechanical parameters of the machine should be taken into account (see Table 1).

**Figure 1:**
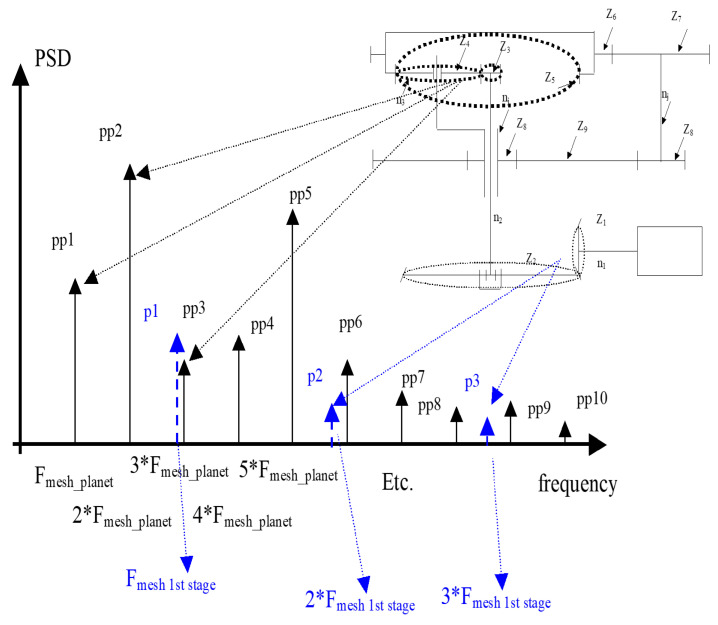
The link between the analysis of frequencies in the spectrum of the signal and the localization of wear in the machine.

The length of the segment comes from the kinematics of the system. In fact, the gearbox is a multi-stage with a bevel stage at the input; the next stage is the planetary stage; and finally, there are three cylindrical stages (see Figure 1). The gearbox is used in the machine working in the mining industry, and it is a critical element of a coal production line. Due to the excavating technology used, the variable properties of excavated material, human and environmental factors, etc., the load of the machine is time-varying. As was said in [17], the variability of the load is related to the variability of gearbox input shaft speed. In the signal set used, the input shaft speed may vary from 940 to 1000 rpm (start up or shut down regimes are not considered). The variability of the speed will affect the mesh frequencies and its harmonics for all stages.

However, by using segmentation, one may neglect speed fluctuation inside the segment, i.e., its influence on the spectral structure, especially the smearing effect. Differences between the speed in segments for the non-loaded and loaded machine may reach even 40–50 rpm and may significantly change the frequency content of the signal. Information about the instantaneous speed taken from the tachometer (the value of speed for each segment) was used for the automatic detection of mesh frequencies.

In a general sense, one may notice that short time frequency analysis is exploited here. By using segmentation, and subsequent spectral analysis for each segment, the feature extraction procedure can be identified as spectrogram estimation (just without overlapping), which is very suitable for non-stationary signals.

To obtain the spectral content of the time series s(n), one may use the discrete Fourier transform (DFT), which decomposes the signal into the family of sinusoidal components with various amplitudes.

As a result, one obtains the distribution of the energy of the signal along the frequency axis. Energy, dissipated by the machine, is one of the parameters describing its condition. Knowledge about the scheme of the machine, frequencies of excitation in the system (related to the rotating parts), allows localizing the change of the condition (one may point out elements in a machine in bad condition; for example, mesh frequency fmesh1 is related to the bad condition of the wheel denoted as z1, z2; see Figure 1).

**Figure 2:**
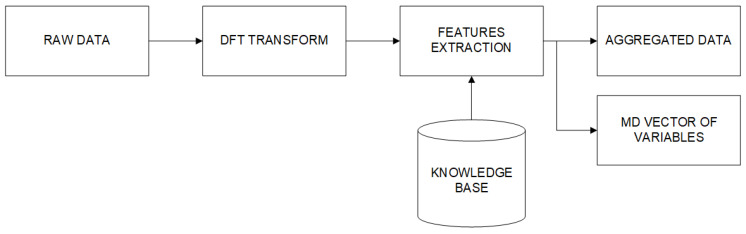
Features extraction procedure for 1D and MD data.

**Figure 3:**
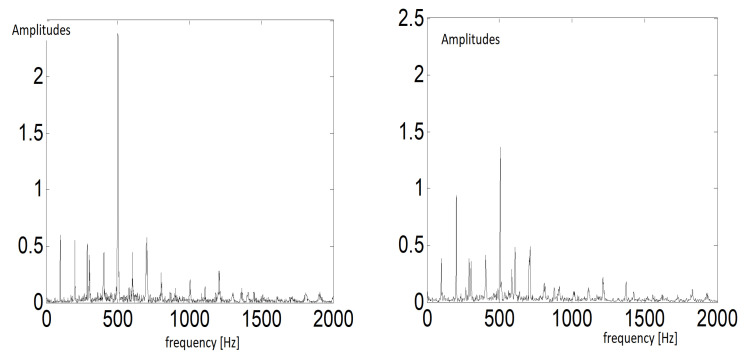
Examples of the spectra (results of spectral analysis)—(**left**): bad condition data, (**right**): good condition data.

The feature extraction procedure is shown in Figure 2. In Figure 3, an example of the spectral analysis results for given segments of vibration data is shown (left: bad condition data, right: good condition data). It is easy to notice the comb of spikes in the spectrum. This family of components is in equal distance related to the planetary mesh frequency (planetary stage) in the gearbox.

The aim of this work is limited to diagnosis (classify data) of the planetary stage extracted by the procedure discussed above in this section.

**Figure 4:**
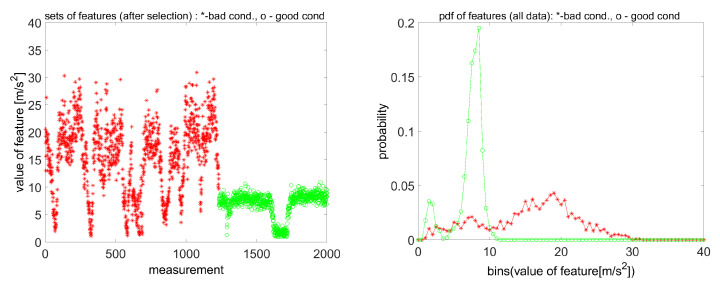
Aggregated feature series for bad and good conditions (**left**) and Histograms of good and bad condition data **(right**)

Using the amplitudes of these components, it is possible to describe the condition of this stage. The general diagnostic rule is very simple: if the energy of dissipation is increasing, it is associated with a change (deterioration) of condition. Bartelmus and Zimroz [17] noticed that non-stationary operating conditions influence the value of such features; thus, in practice, it leads to false alarms (there is no chance to establish an alarm level, i.e., the threshold for the data, because histograms are overlapping (Figure 4). It should be emphasized that many professional systems simply compare the value of the feature with the threshold.

**Figure 5:**
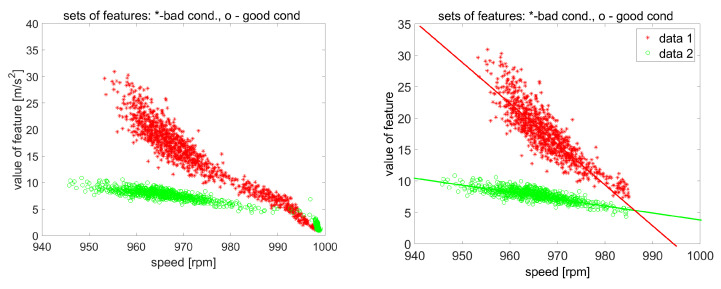
Method of classification in two-dimensional space: (**left**)—whole data, (**right**)—data for a limited speed range only.

In [17], it was proposed to modify the reasoning process by observing both the sum of spectral components (total dissipation energy associated with a given part of the machine) and the operating conditions. Such an approach has allowed the recognition of faultless data measured under proper load conditions, but with a small load or without a load, there is still a serious problem ( Figure 5). It is shown in Figure 5 that the sum of the above-mentioned amplitudes presented as a function of instantaneous input shaft rotational speed is much easier to interpret than a histogram of these sums. It is clear that for lower speed (that is equivalent to a bigger load), the amplitude of feature is greater. For some parts of a speed range, it may even be considered as a linear dependency (as was also suggested by Cempel [23]). Unfortunately, for a speed greater than 995 rpm (small load or unloaded gearbox), the relation between feature-speed reveals serious nonlinearity, and the diagnostic rule that bad conditions generate a greater value of feature than good conditions is not valid. In [17], this speed range was not considered for data classification/recognition.

It is proposed here not to aggregate spectral components, but to exploit the potential of the multidimensionality of the data by taking advantage of the probable multidimensional relation between spectral components. From Figure 5, one may see that in general, this relation for an aggregated feature is non-linear and complicated. We expect that a change of condition and a change of load can influence the structure of the data, but in a different way, and by multidimensional analysis, one may exploit multidimensional information and improve the classification results, even for an unloaded gearbox. This was been investigated in [27]. Therefore, as input data for further classification, we built a matrix of diagnostic data MDD with 15 columns related to the 15 spectral components (where pp1is the amplitude of the planetary mesh frequency (PMF), pp2 is the amplitude of the second harmonic of the PMF, …, pp15 is the amplitude of the 15th harmonic of the PMF).

The final dimension of the diagnostic features matrices is: 1232 × 15 and 951 × 15 for the bad and good condition, respectively.

During the experiments, two gearboxes (the same type) used in a bucket wheel excavator were investigated. Gearbox B with a lifetime B = 20,000 h was described as “designated to major overhaul”. Gearbox A with a lifetime A = 10,000 h was described as a machine in good condition. In the damaged gearbox, visual inspection of the condition done by the manufacturer confirmed the bad condition of the teeth surfaces. After dismantling of the gearbox, which was in operation 20,000 h, the gearbox condition directions for repair were defined: all rolling elements’ bearings have over-limit radial backlash, so they have to be replaced by new ones, and almost all gears should be improved by grinding. Scuffing on the teeth and micro-cracks were also spotted [17].

Algorithm 1 presents an overview of the general learning algorithm.
**Algorithm 1:** General learning algorithm for decision trees with two classes (A and B)1Create a root node v for the decision tree. Let D[v] denote the learning data sample.2If D[v] contains examples of the A class only, mark the node v as terminal, and assign a class label A. Return.3If D[v] contains examples of the B class only, mark the node v as terminal, and assign a class label B. Return.4Try to find the most discriminant attribute and condition on it for the data sample D[v]; otherwise, mark the node v as terminal, and assign a class label represented by most of the data sample D[v]. Return.5Add two edges to the node v with two new nodes v1 and v2. Split the data sample D[v] into two new samples, D[v1] and D[v2], according to the condition found.6Run the algorithm for v1 and D[v1].7Run the algorithm for v2 and D[v2].

It should be noted that despite the significant difference between the lifetime and change of the condition, simple diagnostic methods using energy-based parameters failed due to the variability of the external load.

### 2.2. Decision Trees Classifiers

Decision trees are a classification method in which the classification task is modeled with the use of a set of hierarchical decisions on the feature variables, in the form of a tree [55]. Decision trees gained popularity mainly due to the high efficiency, straightforward interpretation, and clear visualization, compared to other classification approaches [47,48]. A decision tree is an acyclic connected, directed graph where each inner node contains a condition on input objects, outgoing edges correspond to possible values of the state (usually true or false), and each terminal node contains a class label. The decision at a particular node of a tree is related to the condition (split criterion). The condition divides data into two or more subsets. Classification based on a decision tree, constructed and tested earlier on a certain training data sample, begins with checking the condition of the input object in the root node, following the appropriate outgoing edge, checking the condition at the next node, and repeating this until a terminal node with a class label is reached [55].

Algorithm 1 presents an overview of the general learning algorithm.

Terminal nodes result from the stopping criterion. One of the stopping criteria is the situation where all observations in the leaf belong to the same class; however, such an approach may lead to overfitting, in which the model fits the noise in the training dataset. To avoid overfitting, one possibility is to stop the growth of the tree during training [55]. Stopping criteria that can be used in such a case include the maximum depth of the tree, the maximum number of observations in the leaf node, or the maximum number of leaf nodes.

In practice, various discriminant measures for the splitting of attributes can be used. The basic ones are the Gini index and entropy; however, variance reduction or the Chi-squared test can also be applied [48].

The Gini index, also known as Gini impurity, was one of the first measures used in binary trees (CART). Its formula is given below [55]:

$$G\left(v_{i}\right)=1-\sum_{j=1}^{k}p_{j}^{2}$$

where:

$v_{i}$ is the possible value of a particular categorical attribute,

j is the number of classed for the attribute value $v_{i}$, and

$p_{j}$ is the fraction of data points containing attribute value $v_{i}$.

Gini impurity as the splitting criterion should be minimized.

The similar goal as the Gini index is achieved by the entropy criterion; however, it is based on information theory principles [55]. Entropy for the attribute value $v_{i}$ is expressed as follows:

$$E\left(v_{i}\right)=-\sum_{j=1}^{k}p_{j}log_{2}\left(p_{j}\right)$$

In the decision trees classifiers, the information gain criterion is used and is equal to the reduction in the entropy of set S and entropy as a result of the split. Splitting of the nodes is performed due to the maximization of information gain.

More sophisticated classifiers have been proposed, based on random splitting of nodes. One of the examples is the extra tree classifier (also known as extremely randomized trees), in which nodes are split randomly with a random subset of the features selected at every node. That approach was extended with a meta-classifier approach, e.g., random forest or AdaBoost. Their primary assumption relies on the usage of randomization-based ensemble techniques using, e.g., sample bootstrapping to create the set of decision trees [51], which together provide the best classification accuracy.

In our research, we tested several decision tree algorithms: CART with the Gini and entropy criteria (CART-G and CART-E) and random tree (RT, extra tree classifier). We have also used the K-nearest neighbors algorithm (KNN) and meta-classifiers, namely: random forest (RF) and AdaBoost (ADA) for comparison purposes. The detailed results are presented in the next section.

## 3. Results and Discussion

In the experiments, the data sample of 2183 input vectors was used. Each input vector was described by 15 features and a class label. No contradictory input vectors were found in the data under analysis (i.e., vectors with equal values of all 15 features, but with different class labels). In learning classifiers, we used two approaches: first, splitting the dataset into training and test datasets and, second, cross-validation.

In the first approach, the entire dataset was split into a training dataset (65/100) and a test dataset (35/100) randomly. The training dataset was used for training a tree classifiers, and the test dataset was used for validating the constructed classifier. A detailed description of these datasets is provided below:

Training dataset:

Number of observations: 1418

Number of observations of the first class (bad condition): 618 (43.58%)

Number of observations of the second class (good condition): 800 (56.42%)

Test dataset:

Number of observations: 765

Number of observations of the first class (bad condition): 333 (43.53%)

Number of observations of the second class (good condition): 432 (56.47%)

**Figure 6:**
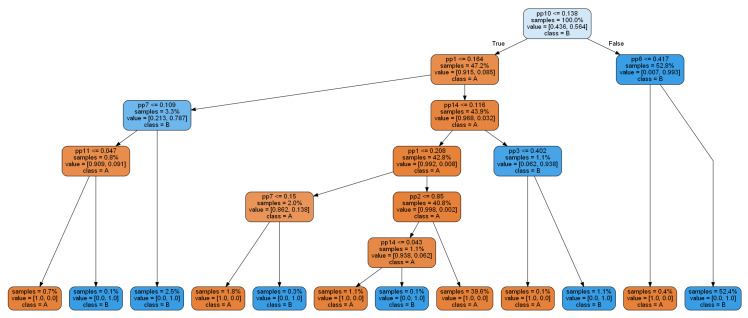
An example of a decision tree, built by the CART algorithm with the Gini criterion based on the training dataset.

The first tree classifier obtained with classic the CART algorithm is presented in Figure 6.

Each non-terminal node contains a condition on one of the 15 features (denoted by pp1, pp2, …, pp15). Each terminal node (leaf) contains a class label (A or B). Running through the decision tree, from its root to one of the leaves, according to the particular values of a given feature vector, leads to a classification of the given feature vector. In this example, the decision tree gives a perfect 100% classification: each leaf corresponds to a data sample of the same class (either A or B). Very often, in practice, such perfect classification of training data does not usually correspond to the perfect classification of test data (unknown during the decision tree construction), sometimes due to the overfitting effect. In our case, classification accuracy for the test dataset was also high and equal to 99.86%. However, the applied algorithm results in too detailed leaves (with a small number of observations); hence. tree generalization is advisable (this will be presented in the latter part of the paper).

In the second approach, the entire dataset was split into 10 folds randomly. One fold was used as the test dataset, while the remaining nine folds were used as the training dataset. The training dataset was used for training a tree classifier, and the test dataset was used for validating the constructed classifier. The procedure was repeated 10 times, each time for the different test datasets, and the results were averaged.

### Comparison of Selected Classifiers

It is well known that different learning algorithms may lead to different decision trees for the same training dataset, as they are based on different foundations. Furthermore, in practice, some hard to define additional constraints and expectations often occur, which cannot be incorporated in the learning algorithm and unambiguously addressed, such as simplicity or a desire for a clear interpretation of the solution returned by the learning algorithm, which makes applying a few training algorithms reasonable and interesting for comparing and studying a few solutions based on different principles. Classification results for the CART-E and RT algorithms are presented in Table 2.

The results obtained during the training phase proved that in general, the decision trees, as knowledge structures, are capable of modeling data related to planetary gearbox condition monitoring, because the efficiency of the decision trees constructed by the proposed algorithm exceeded 99% on the training data, which was a random sample drawn from the entire dataset; therefore, it should have the same characteristic as the entire dataset.

**Figure 7:**
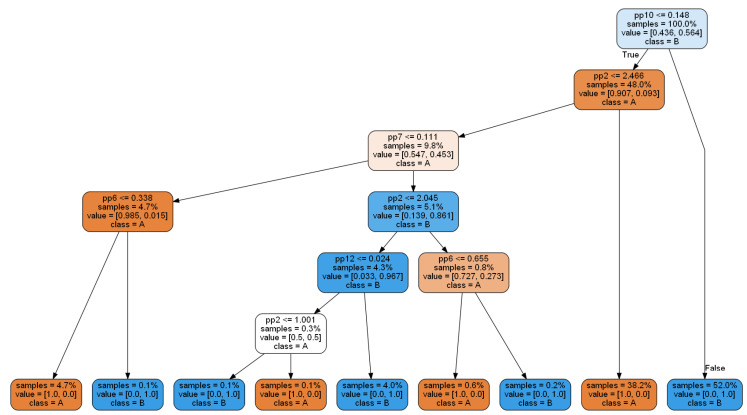
CART-E decision tree.

In the evaluation of selected algorithms, classification accuracy on the test dataset is important. One can observe that the complex RT algorithm as a multi-tree structure has the worst result. The usage of all features in this complex structure did not create the best classifier. The best result was achieved by the CART-E classifier with 100/100 classification accuracy, with only five features included. Its structure is also simpler than the CART-G classifier (Figure 7), leading to nine terminal nodes (CART-G has twelve terminal nodes).

In two CART-based classifiers, we can find similar features, namely: pp10, pp2, pp7, and pp6. Similarly, in both cases, feature pp10 is the most important one.

To generalize the results and to check the objectivity of CART classifiers, we applied the cross-validation approach. Averaged results for classifiers are presented in Table 3.

The CART-E classifier obtained better results than CART-G, also with a smaller standard deviation of the results. We also compared the created decision trees with other classifiers: a simpler one (KNN) and more complex ones (RF and ADA). In Table 4, we remark that the results are better than those of CART-E.

Analyzing the KNN results, we can observe that higher accuracy is related to a small number of neighbors, leading to an overfitting effect. The RF classifier has better results for some number of estimators, mainly for higher values of estimators than CART-E. The ADA classifier did not reach the level of CART-E accuracy.

We can notice that simpler structures as CART trees are capable of achieving comparable or even better results than much more complex structures (RF or ADA). This is especially valuable in the context of expressing the classifier in terms of rules and their implementation in industrial practice.

To avoid the overfitting effect and to generalize the obtained CART-E model, we introduced the stopping criteria during tree training. Firstly, we performed simulations with the following criteria: the maximum depth of the tree, the minimum number of samples in the leaf node, and the maximum number of leaf nodes.

**Figure 8:**
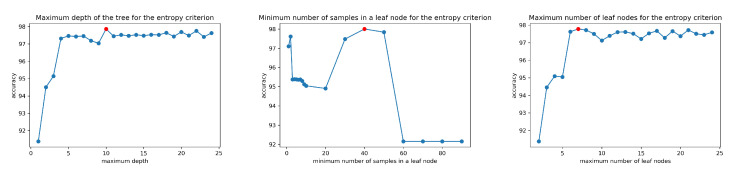
Simulation results for generalization purposes of the CART-E tree: (**left**)-max depth of the tree, (**middle**)-max number of samples in a leaf node, (**right**)-max number of leaf nodes.

The results on the experiments for the optimal size of the CART-E tree are presented in Figure 8 (left-max depth of the tree, middle-max number of samples in a leaf node, right- max number of leaf nodes) and Table 5.

**Figure 9:**
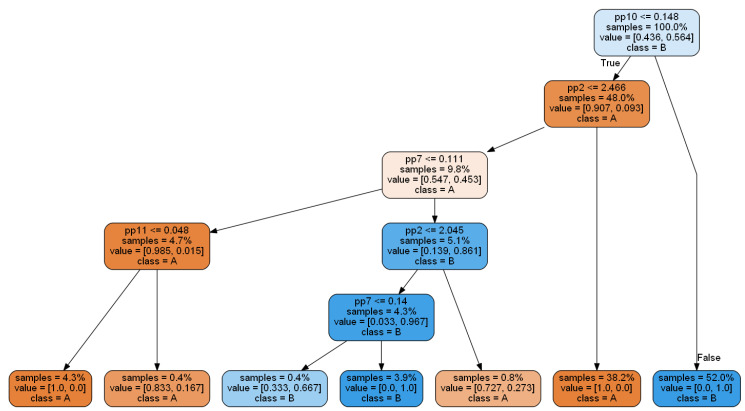
Generalized CART-E decision tree.

In the next step, we set optimal values of the criterion in the learning algorithm. As a result, we obtained the best CART-E tree, presented in Figure 9.

We can observe that the number of terminal nodes decreased to seven. Furthermore, the number of features decreased to four with remaining features: pp10 (with importance 0.7944), pp2 (0.1335), pp7 (0.0694), and the new one: pp11 (0.0026). Correctly classified instances on the test dataset for presented CART-E equal to 99.74%.

The obtained tree classifier can be easily implemented in condition monitoring systems because it can be easily presented as a set of “if-then” rules.

An example of a rule from the CART-E model is presented below:

IF pp10 <= 0.148 and pp2 <= 2.466 and pp7 <= 0.111 and pp11 <= 0.148

THEN class = A

Each rule can also be easily evaluated with a threshold value, expressing its validity. In the case of the exemplar rule, the threshold equals 100%. This means that this rule is always valid. However, we can also find weaker rules in the tree, e.g.: IF pp10 <= 0.148 and pp2 <= 2.466 and pp7 <= 0.111 and pp11 > 0.148 THEN class = A with threshold 83,3%.

Obviously, all rules resulting from the obtained model are valid only for the systems relating to the same type of machine and a similar type of damage (wear, i.e., distributed, not localized).

## 4. Conclusions

A new approach for the diagnosis of the planetary gearbox using spectral features and decision trees is proposed in the paper. As the input features, two datasets with dimension D = 15 and a total number of examples for good and bad conditions bigger than 2000 samples were used. Features were obtained via a spectral representation of the raw signal segmented in the time domain. Each parameter from the single vector was related to the amplitudes of planetary mesh frequency and its harmonics. These datasets were taken from previous papers [17].

The purpose of the paper is to exploit the multidimensionality of the data and compare results to previous approaches when aggregated variables instead of 15D datasets are used.

From the feature extraction point of view, one may notice that data selection has two levels in this paper. The first level is rigid and related to the selection of the components from a spectral representation of the segmented signal (planetary gearbox mesh frequency and its harmonics). It was possible to detect 15 components in the spectrum. The procedure was fully automated.

During the second stage, we assumed that the obtained features might be correlated, and there is no need to use all of them to separate two (good/bad) datasets. By the application of decision trees, we found what features were the most important ones. Therefore, the second level of feature selection comes from decision trees. Apart from the classification of the datasets, we obtained also knowledge about what features are informative from a separation ability point of view, which is very important during implementation in the monitoring system.

In our research, we tested several algorithms, CART with Gini’s index, CART with the entropy measure, and random tree, and compared the obtained results with the KNN classification algorithm, random forest, and AdaBoost.

The application of decision trees allowed us to obtain good results; for some models, there was even a 100% efficiency recorded in the training phase. However, to avoid overfitting, as the next step, we implemented stopping criteria during the training phase to make the tree structure simpler and more general.

As a result of our work, we obtained the CART model based on the entropy criterion with 99.74% classification accuracy on the test dataset.

This study shows that:

- Decision trees can be implemented successfully for diagnosis purposes of the planetary gearbox.
- The application of decision trees enables the modeling of data in a very simple way as a set of hierarchical decisions with information about features’ importance.
- The tree classifier can achieve high efficiency on data classification (in our case, almost 100%), even after generalization.
- The tree classifier, trained on a real dataset, is much simpler than neural network-based classifiers, which is the most important regarding the usage of the results in the industrial applications.
- The tree classifier can be easily implemented in a real-time online monitoring/diagnostic system (especially when online spectral analysis and tracking of several components are commercially available in many systems), and it only requires a transformation of a tree structure into a set of IF-THENrules.
- Each IF-THEN rule can be evaluated with threshold enabling assessment of rule importance for practical usage.

Obviously, for other types of machines, the system should be trained again. It works for a specific type of change of condition, namely wear; for other forms of change of condition, for example for local damages, the proposed technique cannot be used.

Please note that in the paper proposed by Bartelmus and Zimroz [17] for the same data, the efficiency of data recognition was around 80%. Zimroz and Bartkowiak [28] also proposed multivariate procedures (instead of global parameters, they used 15D data space) and got nearly 100% efficiency; however, they used PCA and CDA, which transform data from the original variables to new ones. In the methods presented here, we selected data without processing them, so we did not lose the physical link with the energy of the components.

## Figures and Tables

**Table 1:** Parameters of the system with gearbox: shaft speeds and mesh frequencies.

Shaft Speed	Speed Value (rpm)	Shaft Frequency (Hz)
n1	980	16.33
n2	289.18	4.82
n3	97.80	1.63
narm	22.41	0.37
n4	26.59	0.44
**Mesh Frequency**		**Mesh Frequency Value (Hz)**
f12		294.00
f34		102.26
f67		26.15
f89		8.96

**Table 2:** Results for the selected algorithms. G, Gini; E, entropy.

Algorithm	CART-G	CART-E	RT
Correctly classified instances on the training set (%)	100%	100%	100%
Correctly classified instances on the test set (%)	99.87%	100%	98.95%
Importance of the features	pp10: 0.8361	pp10: 0.7838	pp7: 0.2715
	pp1: 0.0729	pp2: 0.1346	pp13: 0.1704
	pp14: 0.0412	pp7: 0.0633	pp14: 0.1518
	pp7: 0.0298	pp6: 0.0119	pp5: 0.0955
	pp6: 0.0142	pp12: 0.0062	pp3: 0.0717
	pp3: 0.0026		pp6: 0.0597
	pp11: 0.0026		pp11: 0.0534
	pp2: 0.0001		pp4: 0.0284
			pp8: 0.0283
			pp2: 0.0231
			pp1: 0.0179
			pp10: 0.0131
			pp12: 0.011
			pp9: 0.0021
			pp15: 0.0015

**Table 3:** Results of cross-validation approach for CART algorithms.

Algorithm	CART-G	CART-E
Mean accuracy on test sets (%)	96.33	97.75
Standard deviation	0.0854	0.0442

**Table 4:** Results of correctly classified instances for the meta-classifiers and KNN (with mean accuracy on the test datasets). ADA, AdaBoost.

Number of Estimators/Neighbors	KNN	RF	ADA
1	98.53%	95.50%	91.33%
2	98.94%	97.70%	91.05%
3	97.84%	98.85%	93.62%
4	97.98%	94.35%	93.71%
5	97.43%	96.14%	97.01%
6	97.43%	97.79%	97.01%
7	97.24%	97.93%	97.33%
8	97.38%	99.40%	97.29%
9	97.15%	97.79%	97.23%

**Table 5:** Optimal values of CART-E tree construction.

Criterion	Value
maximum depth of the tree	10
minimum number of samples in the leaf node	40
maximum number of leaf nodes	7
